# Different gut microbiota in U.S. formula-fed infants consuming a meat vs. dairy-based complementary foods: A randomized controlled trial

**DOI:** 10.3389/fnut.2022.1063518

**Published:** 2023-01-26

**Authors:** Minghua Tang, Cheng Ma, Eileen M. Weinheimer-Haus, Charles E. Robertson, Jennifer M. Kofonow, Lillian M. Berman, Akbar Waljee, Ji Zhu, Daniel N. Frank, Nancy F. Krebs

**Affiliations:** ^1^Section of Nutrition, Department of Pediatrics, School of Medicine, University of Colorado Anschutz Medical Campus, Aurora, CO, United States; ^2^Department of Statistics, University of Michigan, Ann Arbor, MI, United States; ^3^Division of Gastroenterology, Department of Internal Medicine, Michigan Medicine, Ann Arbor, MI, United States; ^4^Division of Infectious Disease, Department of Medicine, University of Colorado Anschutz Medical Campus, Aurora, CO, United States; ^5^Center for Clinical Management Research, VA Ann Arbor Healthcare System, Ann Arbor, MI, United States

**Keywords:** complementary feeding, gut microbiota, growth, infant, RCT—randomized controlled trial

## Abstract

**Objective:**

This project aimed to evaluate the impact of meat- vs. dairy-based complementary foods on gut microbiota and whether it relates to growth.

**Design:**

Full-term, formula-fed infants were recruited from the metro Denver area (Colorado, US) and randomized to a meat- or dairy-based complementary diet from 5 to 12 months of age. Infant’s length and weight were measured, and stool samples were collected at 5, 10, and 12 months for 16S rRNA gene sequencing and short-chain fatty acids (SCFAs) quantification.

**Results:**

Sixty-four infants completed the dietary intervention (*n* = 32/group). Weight-for-age Z (WAZ) scores increased in both groups and length-for-age Z scores (LAZ) increased in the meat group only, which led to a significant group-by-time interaction (*P* = 0.02) of weight-for-length Z (WLZ) score. Microbiota composition (Beta-diversity) differed between groups at 12 months (weighted PERMANOVA *P* = 0.01) and had a group-by-time interaction of *P* = 0.09. Microbial community richness (Chao1) increased in the meat group only. Genus *Akkermansia* had a significant group-by-time interaction and increased in the dairy group and decreased in the meat group. A significant fold change of butyric acid from 5 to 12 months was found in the meat group (+1.75, *P* = 0.011) but not in the dairy group. Regression analysis showed that Chao1 had a negative association with WLZ and WAZ. Several genera also had significant associations with all growth Z scores.

**Conclusion:**

Complementary feeding not only impacts infant growth but also affects gut microbiota maturation. Complementary food choices can affect both the gut microbiota diversity and structures and these changes in gut microbiota are associated with infant growth.

## Introduction

The role of gut microbiota in human health, including obesity risks, has been examined primarily in adults and animal models (1, 2). Emerging research suggests that early in-life colonization plays a critical role in the establishment and maturation of gut microbiota (3, 4). Early complementary feeding (∼5–12 months of age) represents the time when solid foods are progressively introduced to infants as they no longer rely solely on breastmilk or infant formula. Growth trajectories and shifts in gut microbiota during this period have the potential to program long-term weight, body composition, and disease risks. Emerging research suggests a causal association between gut microbiota and infant growth. A large cohort study (5) identified bacterial species whose proportional representation defined a healthy and mature gut microbiota during the first year of life in Malawian infants. Specifically, deviation from the normal gut microbiota, such as low diversity and absence of certain species, characterized an “immature” gut microbiota that was associated with growth impairment (5). Furthermore, transplanting gut microbiota from stunted infants to germ-free mice also transmitted impaired growth phenotypes in mice (5). Two more recent cohort studies found that disrupted maturation of the gut microbiota, as indicated by low diversity, is associated with slower weight gain in Malawian infants and growth failure in preterm infants (6, 7). Because most of the currently limited evidence was collected in low resource settings and in cohort studies, it is still unclear how complementary foods could affect infant gut microbiota and the potential associations between diet, gut microbiota, and infant growth in controlled trials and in westernized settings.

Although the gut microbiota is greatly influenced by diet, very few studies have addressed the effects of solid foods (complementary foods) on the development of the infant gut microbiota. Our group conducted one of the first controlled feeding studies in breastfed infants on gut microbiota in westernized settings (8) and found that a meat-based complementary diet, compared with a conventional iron-fortified infant cereal-based diet, increased the abundances of certain commensal strains such as the short-chain-fatty-acid-producing clostridia (i.e., *Lachnospiraceae*) from 5 to 9 months of age. This study, although having a small sample size and short duration, demonstrated the potential impact of complementary foods on infant gut microbiota development. Whether these diet-induced gut microbiota changes are associated with infant growth needs further exploration.

We recently completed a randomized controlled feeding study that compared two types of protein-rich foods (meat vs. dairy) as the main source of protein from complementary foods between 5 and 12 months of age on infant growth in formula fed infants (9). We discovered that length-for-age Z score (LAZ, linear growth parameter) increased in the meat group and decreased in the dairy group from 5 to 12 months, which resulted in a significant increase of the parameter weight-for-length Z score (WLZ) and increased risk of overweight in the dairy group (9). However, mechanisms of the differential growth trajectories in response to dairy vs. meat protein remain unclear. Circulating insulin, IGF-1, and IGFBP3, which promote infant weight and length gains, increased from 6 to 12 months but did not differ between groups at 12 or 24 months (9, 10). Although these biomarkers have been suggested as mediators linking protein in infant formula to rapid weight gain (11), the similar increase of these biomarkers between meat and dairy groups in our study did not explain the differential growth patterns between infants consuming meat vs. dairy-based complementary diets. Likewise, analysis of untargeted serum metabolomics (12) also found no significant differences between the meat and dairy groups at 12 months or associations between metabolites and infant growth parameters. The objectives of the current study were to evaluate the impact of complementary foods on infant gut microbiota development and the potential associations between gut microbiota and infant growth. We hypothesized that meat- and dairy-based complementary foods will have differential impact on infant gut microbiota diversity and composition, which will associate with infant growth.

## Materials and methods

### Study design and sample collections

Full term, healthy infants from the metro Denver area (Colorado, USA) who were exclusively formula-fed were recruited and randomized to consume a meat- or dairy-based complementary diet from 5 to 12 months of life, with meat- or dairy as the primary source of protein from complementary foods (9). Total protein intake during the intervention was targeted at 15% of total energy consumption or 3 g/kg/day. The meat group consumed pureed beef, pork, and poultry (provided), and the dairy group consumed yogurt, cheese, and whey protein powder (provided). Upon enrollment, to standardize the formula exposure, the same cow-milk-based formula was also provided by study investigators to all participants. The amount of formula consumed between groups did not significantly differ during the intervention (9). Both groups consumed comparable amounts of protein and minimal protein from the assigned alternative. Participants’ length and weight were assessed at baseline (5 months), at end of the intervention (12 months) and at monthly home visits. Stool samples were collected at 5, 10, and 12 months of age. Soiled disposable diapers fitted with biodegradable liners were collected from participants’ home and transferred back to the laboratory on dry ice. Clinical grade gloves and sterile fecal swabs were used to avoid microbial contamination while collecting the stool samples. Samples were stored at –80 degree C until analyzed. This study was approved by the Colorado Multiple Institutional Review Board and was registered at ClinicalTrials.gov (NCT02142647).

### Microbiome analysis

#### 16S amplicon library construction

Bacterial profiles were determined by broad-range amplification and sequence analysis of 16S rRNA genes following our previously described methods (13, 14). In brief, DNA was extracted from 25 to 50 mg of stool using the QIAamp PowerFecal DNA kit (Qiagen Inc., Carlsbad, CA), which employs chemical and mechanical disruption of biomass. PCR amplicons were generated using barcoded (15) primers that target approximately 450 basepairs of the V3V4 variable region of the 16S rRNA gene (338F: 5′ACTCCTACGGGAGGCAGCAG and 806R: 5′ GGACTACHVGGGTWTCTAAT) (16, 17). PCR products were normalized using a SequalPrep™ kit (Invitrogen, Carlsbad, CA) and then pooled. The amplicon pool was partially lyophilized to reduce its volume then purified and concentrated using a DNA Clean and Concentrator Kit (Zymo, Irvine, CA). Pooled amplicons was quantified using a Qubit Fluorometer 2.0 (Invitrogen, Carlsbad, CA). Illumina paired-end sequencing was performed following the manufacturer’s protocol on the MiSeq platform using a 600 cycle version 3 reagent kit and versions v2.4 of the MiSeq Control Software.

#### Analysis of Illumina paired-end reads

Illumina Miseq paired-end reads were aligned to human reference genome hg19 with bowtie2 and matching sequences discarded (18, 19). As previously described (20), following demultiplexing, each pair of forward and reverse fastq reads was merged into a single sequence using phrap (21, 22) and read pairs that could not be merged were discarded. The resulting sequences were trimmed over a moving window of 5 nucleotides until average quality met or exceeded 20. Trimmed sequences with more than 1 ambiguity or shorter than 350 nt were discarded. Potential chimeras identified with Uchime (usearch6.0.203_i86linux32) (23) using the Schloss (24). Silva reference sequences were removed from subsequent analyses. Sequences were aligned and classified using SINA (1.3.0-r23838) (25) and the 418,497 bacterial sequences in Silva 115NR99 (26) as reference configured to yield the Silva taxonomy. Taxonomic assignment by SINA used the lowest common ancestor approach with default parameters. Closed-reference operational taxonomic units (OTUs) were produced by binning sequences with identical taxonomic assignments; taxonomic bins for sequences that were not classified to the genus level are appended with the label “_uncl” (e.g., *Lachnospiraceae_uncl*). This process generated a median of 81,387 sequence/sample (IQR: 49,385–119,001) for 168 samples. The software package Explicet (v2.10.5) (27) was used for microbial diversity analysis.

### Short-chain fatty acid assessment

Fecal short-chain fatty acid (SCFAs) analysis was conducted at the Mayo Clinical Metabolomics Core lab. SCFA were quantitated *via* GCMS with a few modifications. Briefly, 50 μl fecal water was added to a tube containing internal standard (2-ethylbutyric acid) in HCl. One milliliter of dichloromethane (DCM) was used to extract SCFA from the mixture. The extract was derivatized with N-Methyl-N-tert-butyldimethylsilyltrifluoroacetamide (MTBSTFA) prior to analysis on GCMS. Concentrations of acetic acid (m/z 117.0), propionic acid (m/z 131.1), isobutyric acid (m/z 145.1), butyric acid (m/z 145.1), isovaleric acid (m/z 159.1), valeric acid (m/z 159.1), isocaproic acid (m/z 173.2), and hexanoic acid (m/z 173.2) were measured against 11-point calibration curves that underwent the same derivatization (28, 29).

### Statistical approach

The software packages R (v3.6.3) (30) and Explicet (v2.10.5) (27) were used to analyze and visualize data. Values are presented as mean ± SD for continuous variables. Participants’ demographics and growth Z scores between the meat and dairy groups were compared using independent *t-*tests. Nominal *p* < 0.05 was considered significant between groups. Differences in overall microbiota composition (i.e., beta-diversity) were assessed through permutational ANOVA (PERMANOVA) with the Aitchison dissimilarity index applied to sequence count data (31, 32). We entered sequencing batch, diet, age, and the diet × age interaction term into PERMANOVA models, while subject IDs were included as covariates in longitudinal PERMANOVA tests. PERMANOVA *p*-values were inferred through 10^6^ label permutations and type 2 (i.e., marginal) *p*-values are reported. Principal coordinates analysis (PCoA) was carried out using Aitchison dissimilarities and the *wcmdscale* function in the *vegan* R package (32).

Time-series analyses of (1) alpha-diversity indices (i.e., Chao1, Shannon H, Shannon H/Hmax) and (2) individual taxa were conducted using the *lmer* function of the *lme4* R package (33). As fixed effects, we entered sequencing batch, diet, age, and the diet × age interaction term into the models, while subject IDs were included as random effect intercepts. If a diet × age interaction term was not significant (the FDR-corrected *p*-value was > 0.1), the model was re-run after removing the interaction term. Tests performed between dietary groups at individual ages were conducted by simple linear models (R *lm* function) with dietary group as main effect. For analysis of individual taxa, we first used ALDEx2 (34) to estimate the distribution of taxa in each sequence library through 500 Dirichlet Monte Carlo re-samplings of sequence count data, followed by centered log-ratio (CLR) transformation with all features used as the denominator (aldex.clr function). Each re-sampled dataset was then subjected to linear mixed effects modeling with *lmer*; beta estimates and *p*-values were averaged across replicates (34, 35). To minimize the number of statistical tests performed, we limited this analysis to the 50 taxa with the highest relative abundances, which collectively accounted for > 99% of the total sequences in the dataset. Either nominal or FDR-corrected type 2 (i.e., marginal) *p*-values are reported, as indicated in the text and figures. Associations between the relative abundances of bacterial taxa, alpha diversity, and growth Z scores were assessed using stepwise regression to select genera (changes 12–5 months) that have significant linear relationship with the change of WAZ, LAZ, and WLZ.

## Results

### Subjects

A total of 64 infants completed the dietary intervention (*n* = 32 per group) with weight and length data from 5 to 12 months of age. Overall, 59 stool samples were collected at baseline (5 months), 52 at 10 months, and 57 at 12 months (Supplementary Figure 1). Table 1 summarized growth Z scores of those participants from whom stool samples were successfully collected at 5, 10, and 12 months. There were no significant differences between groups for birth length, sex, maternal BMI, or maternal education. Mothers were, on average, overweight, as defined by BMI between 25 and 29.9 (pre-pregnancy BMI). Consistent with the report of growth Z scores in the parent cohort (9), during the intervention, there was a significant group-by-time interaction (*P* = 0.001) of length-for-age Z score (LAZ) from 5 to 12 months, indicating that LAZ increased in the meat group compared to the dairy group. WAZ increased among all participants from 5 to 12 months. Changes in WAZ and LAZ led to a significant group-by-time interaction (*P* = 0.02) of WLZ. Two participants had antibiotics during the intervention (one from each group).

**TABLE 1:** Subject characteristics and growth Z scores^a^.

	Meat group	Dairy group	*P*-value
Birth weight (kg)	3.30 ± 0.41	3.32 ± 0.39	0.45
Maternal BMI^b^	28 ± 6	28 ± 7	0.88
Male (female)	16 (16)	16 (16)	
5 months LAZ	−0.19 ± 0.86	−0.30 ± 1.02	0.63
5 months WAZ	−0.03 ± 0.69	−0.14 ± 0.82	0.59
5 months WLZ	0.08 ± 0.79	0.04 ± 0.66	0.80
12 months LAZ	0.14 ± 0.90	−0.60 ± 0.91	0.002
12 months WAZ	0.40 ± 0.74	0.39 ± 0.78	0.99
12 months WLZ	0.33 ± 0.48	0.95 ± 0.90	0.04

^a^Mean ± SD, ^b^Independent Student’s *t*-test.

### Gut microbiota diversity

**FIGURE 1:**
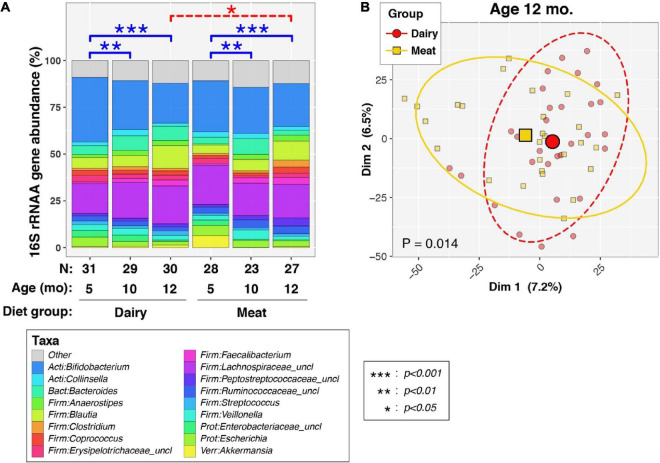
Effects of diet and age on overall composition of infant fecal microbiota. **(A)** Barcharts show average percent relative abundances (%RA) of predominant taxa, stratified by age and dietary group. Taxa with %RA less than 2% were collapsed into the “Other” category to simplify the figure. Differences in bacterial profiles (beta-diversity) between ages and diet groups were assessed by weighted permutational ANOVA (PERMANOVA) tests, which applied the Aitchison dissimilarity index to sequence count data as a measure of beta-diversity. *P*-values were calculated through 10^6^ label permutations. Solid blue brackets and asterisks (see box insert) denote *p*-values for within-group, longitudinal PERMANOVA tests between ages, which included subject IDs as covariates. Dashed red brackets and asterisks (see box insert) denote *p*-values for denote cross-sectional PERMANOVA tests of diet groups at each age. **(B)** Principal coordinates analysis (PCoA using Aitchison dissimilarity scores) of infants at 12 mo. of age. Small symbols represent individual subjects. Large symbols represent group means of PCoA scores along the first and second axis. Ellipses mark 95% confidence intervals. A PERMANOVA test indicated significant between-group differences in beta-diversity (*P* = 0.014), as also indicated in **(A)**.

Overall, all 168 valid stool samples generated > 10,000 16S sequences/sample and were included in the microbiome analysis. The Good’s index of each sequence library (i.e., sample) was > 99%, indicating that the depth of sequencing was sufficient to represent the biodiversity in the specimens. We first examined the effects of age/time and dietary intervention group on the overall structure of the fecal microbiome (i.e., beta-diversity, assessed by PERMANOVA; Figure 1). As expected, age was significantly associated with beta-diversity for all infants taken together (*P* < 1e-06), or when stratified into Dairy (*P* < 1e-06) and Meat groups (*P* = 7.3e-05). Age-dependent differences in microbiota were observed in both dietary groups between 5 and 10 mo. (*P* < 0.01 for each diet group) and between 5 and 12 mo. (*P* < 0.001 for each diet group), whereas no significant differences were apparent between 10 and 12 mo. (*P* > 0.05 for each diet group).

At both 5 and 10 mo., no differences in beta-diversity were observed between the Meat and Dairy groups (*P* > 0.05 at both timepoints). However, at 12 mo., significant differences were observed between Meat and Dairy groups (Figure 1A, *P* = 0.014). A PCoA plot showed some clustering of infants by diet group (Figure 1B), though much overlap in groups was apparent. Finally, a PERMANOVA analysis of all infants modeling diet group, age, and including a group-by-age interaction term found some support for an interaction between diet group and age [P (group × age) = 0.090].

**FIGURE 2:**
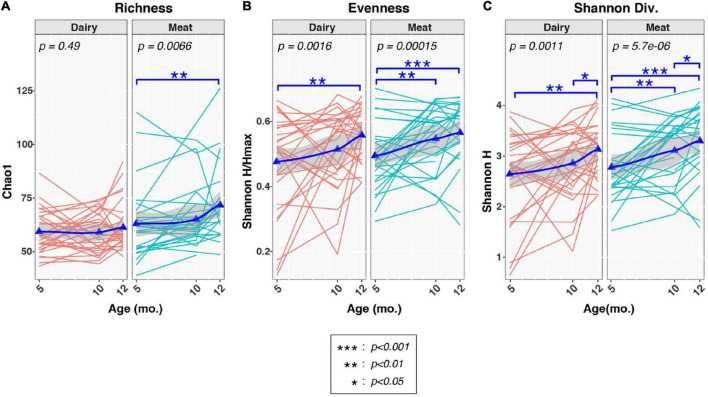
Effects of diet and age on alpha-diversity measures of infant fecal microbiota. Plots show distributions of three alpha-diversity indices [**(A)**: richness, **(B)**: evenness, **(C)**: Shannon Diversity] from 5 to 12 months (mo.) of age, stratified by Diet group. Thin lines indicate changes in alpha-diversity indices over time for individual infants. Triangles represent mean values at each age. Thicker lines represent locally estimated scatterplot smoothing (LOESS), while the shading surrounding each LOESS line represents 95% confidence intervals. Changes in alpha-diversity across time were evaluated in each group by linear mixed-effects models (*lme4* R package), with age modeled as a fixed effect and subject ID used as grouping factor. Overall *p*-values for associations of alpha-diversity with age across each time-series are indicated by the text in each plot. Tests between pairs of ages are indicated by asterisks (see box insert). Analyses of group-by-age interactions are not shown, but produced *p*-values of 0.073 (richness), 0.93 (evenness), and 0.71 (Shannon diversity).

We next examined the effects of age and diet group on three common measures of alpha-diversity (Figure 2). Group-by-age interactions were suggested for richness [P (group × age) = 0.073], but not evenness [P (group× age) = 0.93], or Shannon diversity [P (group× age) = 0.71], indicating that dietary group had limited effect on the trajectory of the overall alpha-diversity through time. In contrast, longitudinal increases in richness, evenness, and Shannon diversity were observed to varying degrees in both Dairy and Meat groups. As with beta diversity, significant increases in alpha diversity were observed mainly in comparisons of 5 vs. 10 months and 5 vs. 12 months, whereas more modest increases in alpha diversity were noted between 10 and 12 mo. Between-diet group analyses of alpha-diversity at each age found that Dairy and Meat groups differed only in richness at 12 months (*p* = 0.0023; data not shown).

Finally, we examined the effects of dietary group and age on the relative abundances of individual taxa. The four most abundant phyla at all three time points were Firmicutes, Actinobacteria, Bacteroidetes, and Proteobacteria, which accounted for over 97% of the overall bacteria abundance (Table 2). None of the phyla differed between diet groups at 5 months. The abundances of Firmicutes increased over time, while Actinobacteria and Proteobacteria decreased, without significant differences between groups (Table 2).

**TABLE 2:** Relative abundance (%) at phylum level by group at 5, 10, and 12 months^a^.

	Groups	Actinobacteria^b^	Bacteroidetes	Firmicutes^b^	Proteobacteria^b^
5 months	Meat	34.75 ± 23.65	5.45 ± 7.51	46.46 ± 23.54	9.40 ± 10.34
	Dairy	32.17 ± 24.74	6.54 ± 10.47	48.36 ± 23.04	9.15 ± 16.54
10 months	Meat	27.39 ± 21.50	10.97 ± 16.01	54.60 ± 19.84	5.91 ± 9.43
	Dairy	31.85 ± 24.28	9.33 ± 13.88	50.91 ± 21.05	6.21 ± 12.13
12 months	Meat	23.91 ± 18.54	5.63 ± 11.16	65.17 ± 18.24	3.23 ± 5.80
	Dairy	23.71 ± 16.61	9.11 ± 11.61	61.84 ± 16.80	4.91 ± 4.77

^a^Mean ± SD.

^b^Significant effect of time: *P* = 0.014 for Actinobacteria, *P* = 0.000004 for Firmicutes, *P* = 0.04 for Proteobacteria.

**FIGURE 3:**
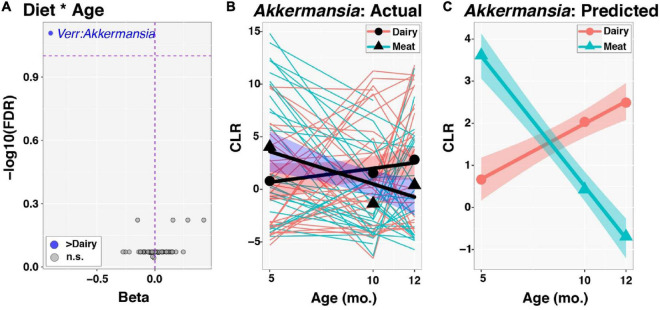
Time-series analysis of individual taxa: Modifying effects of diet. Group by age interactions in the development of individual taxa were evaluated through linear mixed effects modeling, as described in the text. Centered log-ratio (CLR) transformed sequence count data for taxa were included in models as outcome variables. **(A)** Plot of regression beta coefficients (*x*-axis) vs. −log10 transformed, FDR-corrected *p*-values (*y*-axis) from analysis of Diet by Age interactions (Diet * Age). The Dairy group served as the reference, such that taxa that had significantly greater increases in relative abundance through time (FDR < 0.1) in the Dairy group had negative beta coefficients and are displayed to the left and colored blue. Only *Akkermansia* had an FDR-corrected *p*-value < 0.1. **(B)** *Akkermansia* dynamics from 5 to 12mo. of age. Thinner lines represent trajectories for individual infants, color-coded by Diet group. The *x*-axis and *y*-axis specify infant age in months and CLR values for *Akkermansia*, respectively. Circles and triangles represent mean values for each Diet group and age. Thicker lines are fitted values from linear models, while shaded ribbons indicate 95% confidence intervals. **(C)** Predicted CLR values for *Akkermansia* following linear mixed effects modeling. Symbols are as described in **(B)**.

**FIGURE 4:**
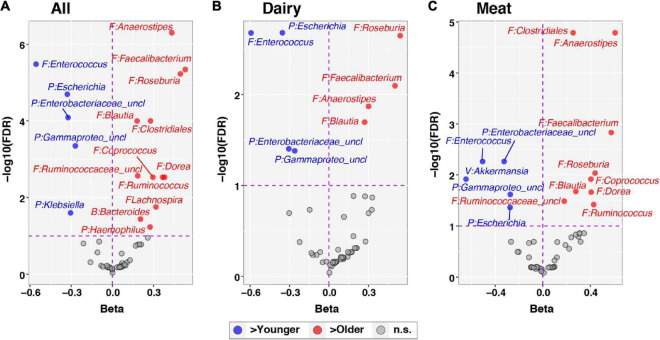
Differential abundance of individual taxa across age. The development of individual taxa with age was evaluated through linear mixed effects modeling, as described in the text. Centered log-ratio transformed sequence count data for taxa were included in models as outcome variables. The panels show results for all subjects, adjusting for Diet group **(A)** and stratified by Dairy **(B)** and Meat **(C)** groups. Each panel plots regression beta coefficients (*x*-axis) vs. –log10 transformed, FDR-corrected *p*-values. Taxa significantly positively correlated with age (FDR < 0.1) have positive beta coefficients and are plotted to the right in red; conversely, taxa negatively correlated with age (FDR < 0.1) have negative beta coefficients and are plotted to the left in blue. Non-significant taxa (FDR > 0.1) are shown in gray. Taxa names are preceded by abbreviated phylum names: B, Bacteroidetes; F, Firmicutes; P, Proteobacteria; V, Verrucomicrobia.

At the genus level, *Akkermansia*, of the phylum Verrucomicrobia, was the only taxon with a significant group-by-time interaction (FDR corrected *p*-value = 0.078; Figure 3A). This genus increased in relative abundance with age in the Dairy group but fell in abundance with age in the Meat group (beta = −0.90, Figures 3B, C). The taxa *Lachnospiraceae_uncl* and *Christensenellaceae_uncl* also had nominal *p*-values < 0.05 for the group × age interaction and beta coefficients of −0.15 and 0.42, respectively. No taxa differed in abundance by diet group after adjusting for age (i.e., all taxa had FDR corrected *p*-values > 0.1 for the main effect of Diet), as expected since infants were randomized to Diet groups. In contrast, 17 taxa were associated with age among all infants after adjusting for Diet group (Figure 4 and Supplementary Figure 1). Of the 5 differentially abundant taxa belonging to the phylum Proteobacteria, 4 decreased in abundance with age. In contrast, 10 of 11 differentially abundant Firmicutes taxa were positively associated with age (Figure 4A and Supplementary Figure 2).

### Short-chain fatty acid

Fecal SCFAs were assessed both qualitatively and quantitatively. A significant fold change of butyric acid from 5 to 12 months was found in the meat group (+1.75, *P* = 0.011) but no change was detected in the dairy group. However, the quantitative analysis did not reveal a significant effect of time or group. Table 3 showed that absolute quantities of fecal SCFAs at 5, 10, and 12 months between groups did not differ.

**TABLE 3:** Fecal short-chain fatty acid concentrations (nmol/g) between groups^a^.

Time	Group	Acetic acid	Butyric acid	Isobutyric acid	Propanoic acid	Valeric acid	Isovaleric acid
5 months	Meat (*n* = 28)	31.9 ± 16.7	3.2 ± 2.4	3.6 ± 3.1	7.3 ± 6.0	0.2 ± 0.4	2.4 ± 2.4
	Dairy (*n* = 31)	35.0 ± 18.7	4.8 ± 5.5	4.8 ± 5.2	9.6 ± 8.5	0.4 ± 1.1	3.7 ± 4.4
10 months	Meat (*n* = 23)	37.5 ± 15.7	5.2 ± 4.3	3.6 ± 2.4	7.1 ± 4.8	0.4 ± 0.7	3.1 ± 2.2
	Dairy (*n* = 29)	35.5 ± 22.9	5.1 ± 3.7	4.4 ± 3.1	8.7 ± 6.2	0.4 ± 0.6	4.6 ± 3.4
12 months	Meat (*n* = 27)	35.0 ± 19.0	5.6 ± 4.1	4.6 ± 2.9	8.5 ± 5.2	0.4 ± 0.6	3.5 ± 2.6
	Dairy (*n* = 30)	35.2 ± 16.1	5.7 ± 4.1	6.4 ± 5.5	10.8 ± 6.8	0.6 ± 1.1	5.7 ± 5.5

^a^Mean ± SD.

### Associations between gut microbiota composition and infant growth Z scores

We also analyzed the potential association between gut microbiota composition and infant growth Z scores. Regression analysis using alpha diversity indicator Chao1 as the predictor of changes of WLZ (12–5 months) showed a nominally significant negative regression model: WLZ = –0.010 * Chao1 + 1.309 (*P* = 0.045). Chao1 was marginally negatively associated with WAZ: WAZ = −0.007*Chao1 + 0.109 (*P* = 0.10). Table 4 lists genera (changes 12–5 months) that had a significant regression coefficient with the changes of WAZ, LAZ, and WLZ (12–5 months).

**TABLE 4:** Regression analysis of genera changes (predictor) and growth Z scores changes from 5 to 12 months of age.

a. ΔWAZ					
**Variable**	**Coefficient**	***P*-value**	**Variable**	**Coefficient**	***P*-value**
Gardnerella	0.138	2.65E-11	RC9_gut_group	0.076	1.05E-05
Salmonella	0.127	3.49E-10	Porphyromonas	0.063	2.11E-05
Leuconosto	0.147	5.00E-09	Sarcina	–0.049	2.75E-05
Acidaminococcus	–0.115	2.54E-08	Thalassospira	–0.053	4.08E-04
Dialister	–0.076	3.26E-08	Arthrobacter	0.061	4.50E-04
Ruminococcus	0.060	1.05E-07	Granulicatella	–0.043	7.86E-04
Flavonifractor	0.082	6.80E-07	Asteroleplasma	–0.110	4.00E-03
(Intercept)	0.286	1.05E-06	Proteiniphilum	–0.065	4.57E-03
Haemophilus_	0.040	2.86E-06	Treponema	–0.054	9.27E-03

**b. ΔLAZ**					

Robinsoniella	0.126	4.67E-06	Anaerofustis	0.045	1.16E-04
(Intercept)	–0.478	1.91E-12	Peptostreptococcus	0.065	1.23E-04
Asteroleplasma	–0.369	3.16E-11	Collinsella	0.026	2.95E-04
Desulfovibrio	0.094	4.32E-11	Phascolarctobacterium	–0.027	3.28E-04
Geobacillus	–0.220	1.38E-09	Corynebacterium	0.028	1.34E-03
Lachnospira	0.050	5.83E-08	Christensenella	–0.078	3.15E-03
Slackia	–0.063	7.87E-08	Anaerovibrio	0.032	1.36E-02
Neisseria	0.082	9.48E-07	Holdemania	–0.015	1.58E-02
Paraprevotella	0.156	1.32E-06	Enterobacter	0.017	1.75E-02
Lactococcus	0.037	3.53E-06	Peptococcus	–0.021	1.94E-02
Catenibacterium	–0.061	3.64E-06	Dolosigranulum	–0.035	4.89E-02

**c. ΔWLZ**					

Akkermansia	0.092	1.89E-12	Lachnoanaerobaculum	0.091	3.56E-04
Bifidobacterium	–0.225	9.38E-08	Asteroleplasma	–0.193	4.49E-04
Gardnerella	0.135	5.32E-07	Roseburia	–0.056	5.63E-04
Eggerthella	–0.103	6.40E-07	Abiotrophia	0.093	2.50E-03
Turicibacter	0.077	1.14E-06	Dialister	–0.042	8.20E-03
(Intercept)	0.515	1.18E-06	Veillonella	–0.055	1.14E-02
Finegoldia	0.089	2.01E-05	Dolosigranulum	–0.080	2.38E-02
Negativicoccus	–0.114	2.20E-05	Butyricimonas	–0.070	2.58E-02
Treponema	–0.134	2.63E-05	Elusimicrobium	–0.055	3.80E-02
Gemella_	–0.077	4.63E-05	Mitsuokella	0.068	4.67E-02

## Discussion

In this study, we compared gut microbiota community structure at 5, 10, and 12 months of age in full-term, formula-fed infants randomized to consume meat- or dairy-based protein-rich foods for a complementary diet, in parallel with clinical growth data. Our results suggest that consuming complementary foods impacted infant gut microbiota development, which is partially dependent upon the complementary diet composition. We also presented the potential associations between gut microbiota and infant growth parameters. This is one of the first studies that evaluated the impact of protein-rich complementary foods on infant gut microbiota, in a longitudinal, randomized controlled trial. In terms of gut microbiota alpha diversity, we found gut microbiota richness increased in infants consuming a meat-based complementary diet from 5 to 12 months.

Although alpha diversity tends to be low in young infants, some studies found that it increased with solid food introduction in infants (36, 37). Increased alpha diversity may be interpreted as increased microbial stability and progression toward a more mature gut microbiota (5), and some emerging research suggests that low diversity may be associated with infant growth failure (6). However, another recent cohort study found that infants at risk of overweight at 12 months had a higher richness at 3–4 months (38). In that study (38), participants with a higher gut microbiota richness at 3–4 months were also formula-fed. It would be difficult to differentiate the effects of formula-feeding vs. higher alpha diversity on the risk of overweight, especially when formula-feeding itself is reported to associate with higher alpha diversity (39, 40). Besides mode of feeding, delivery mode is also reported to affect infant gut microbiota, although its effect may not persist beyond 6 months of age as some research reported (41). In our study, all participants were exclusively formula-fed upon enrollment and mode of delivery did not affect the 5-month gut microbiota composition or diversity. If increased alpha diversity is part of the gut microbiota maturation process, a meat-based complementary diet appeared to foster a more optimal maturation during later infancy.

A unique change of gut microbiota taxa was observed: *Akkermansia* had a significant group-by-time interaction and increased in the Dairy group while decreased in the Meat group overtime. *Akkermansia muciniphila* as the dominating species of genus *Akkermansia* and phylum *Verrucomicrobiota* (42), has been shown in numerous studies to be protective against obesity, type 2 diabetes and other metabolic diseases (43). In infants and children, low abundance of *Akkermansia* appeared to associate with rapid weight gain in observational studies (44, 45), while low *Akkermansia* abundance was accompanied by low richness (Chao1) (44). Interestingly, the present study observed both a decrease of *Akkermansia* abundance and an increase of richness (Chao1) in the Meat group overtime. It is unclear how these two observations together affect infant linear growth and risk of overweight. As discussed previously (9), the greater length gain in the Meat group is hard to interpret as positive or negative growth trajectory in the long-term. Note that although there was a group-by-time interaction of *Akkermansia* abundance, it was higher in the Meat group compared to Dairy at baseline. This could potentially influence the changes overtime. Future research to evaluate the relation of *Akkermansia* and linear growth is warranted.

The effects of types of protein-rich foods on gut microbiota are still being explored. One animal study (46) compared meat-, dairy-, and plant-protein extracts and found *Ruminococcaceae* was one of the characteristic bacteria in rats fed with meat proteins. One RCT of Canadian infants found that consumption of meat-based or cereal and fruit-based complementary diet increased microbial alpha diversity (Chao1), compared with a cereal only complementary diet (37). Another study from our group in 6 to 9 month-old breastfed infants showed that compared with a low-protein, cereal/plant-based complementary diet (9% energy from protein), a high-protein, meat-based diet (17% energy from protein) increased the abundance of SCFA (butyrate)-producing *Lachnospiraceae* (9). That study (9) used iron-fortified infant cereal so both protein and iron intakes differed between the high- vs. low-protein groups. Another study showed a fourfold increase in relative butyrate levels from 6 to 12 months of age, representing the transition from infant to adult-like gut microbiota (47). In the current study, although untargeted analysis showed significant fold change of SCFAs, quantitative SCFA analysis did not show significant differences either over time or between groups, possibly due to the relatively small sample size and large variance to detect potential differences. The Meat group also had a lower SCFA level at baseline, although not statistically different from the Dairy group.

Recent progress in gut microbiota research suggests it may directly influence linear growth. At 12 months, the Meat and Dairy group had different bacterial profiles, as reflected by both beta diversity (Figure 1B) and individual taxa comparisons (Figure 4). Specifically, *Ruminococcus* was enriched in the Meat group. *Ruminococcus* is a potent SCFA producer and also related to infant linear growth. A cohort study (5) identified *Ruminococcus gnavus* and *Clostridium symbiosum* as the two major growth-discriminatory species and the abundance of which dictates length trajectories in growth-impaired human infants and mice. In the current study, none of the participants was considered growth impaired or at risk of stunting, but the Dairy group had a significant increase in WLZ (parameter of overweight risk) due to a decline in LAZ over the intervention period. We found a negative association between microbiota richness (Chao1) and WLZ. Although *Ruminococcus* was enriched at 12 months in the Meat group, regression analysis did not find a significant association between *Ruminococcus* and WLZ. The regression model showed a positive relation of the changes of *Ruminococcus* and WAZ. It is possible that different environments between the Malawi trial (5) and our study, such as the risk for stunting vs. risk for overweight in developing and developed countries, respectively. The environment of the current study (metro Denver area) and the abundant energy and nutrient intakes could potentially lead to WAZ increase. Among the genera that were identified as a significant predictor (independent variable) of LAZ, *Lachnospira* and *Collinsella* were the only two among the top 35 most abundant genera or with a relative abundance over 0.1%. Both *Lachnospira* and *Collinsella* had a positive relation with LAZ. In addition, genera *Bifidobacterium* and *Roseburia* had a negative association with the increase of WLZ, suggesting that these potential probiotics may contribute to lower overweight risks in infants. It is worth noting that WLZ is derived from WAZ and LAZ. In the current study, the increase of WLZ was due to a decrease in LAZ, and no direct association was found between *Bifidobacterium* and *Roseburia* and LAZ. Future research needs to investigate the potential mediating effect of gut microbiota in infant linear growth.

Ten percent or more of the ingested protein can reach the colon, and the amount is at least partially dependent on protein quality (48). Different protein quality between meat, dairy, and plant may result in differing availabilities of dietary proteins to gut microbiota. Amino acid compositions for various protein sources also differ. For example, dairy has a higher amount of branched-chain amino acids than meat and plant (49). Furthermore, undigested protein and amino acids in the colon may serve as an additional substrate for SCFA production besides non-digestible carbohydrates (50). These differences may contribute to the differential impact of protein on gut microbiota structure. Although we did not observe significant changes of SCFAs over time or between groups, it may be due to the relatively small sample size in detecting such changes. Some other limitations of this study include having formula-fed infants only with no breastfeeding reference group and infrequent microbiota sample collections.

Overall, our findings suggest that complementary feeding is an important developmental phase not only for infant growth but also gut microbiota maturation. Complementary food choices could affect both the gut microbiota diversity and community structures, which associated with infant growth. These effects could pose long-term impact which merits further investigation using randomized controlled trials.

## Data availability statement

The datasets presented in this study can be found in online repositories. The names of the repository/repositories and accession number(s) can be found below: https://www.ncbi.nlm.nih.gov/sra/PRJNA884688.

## Ethics statement

The studies involving human participants were reviewed and approved by the Colorado Multiple Institutional Review Board (COMIRB). Written informed consent to participate in this study was provided by the participants’ legal guardian/next of kin.

## Author contributions

MT and NK designed research. MT and LB conducted the experiments and data collection. CR, JK, and DF analyzed the stool samples. MT, CM, EW-H, AW, JZ, CR, and DF analyzed the data. All authors wrote and approved the manuscript.

## References

[1] Turnbaugh P Hamady M Yatsunenko T Cantarel B Duncan A Ley R A core gut microbiome in obese and lean twins. *Nature.* (2009), 457:480–4. 10.1038/nature07540 19043404 PMC2677729

[2] Turnbaugh P Ley R Mahowald M Magrini V Mardis E Gordon J. An obesity-associated gut microbiome with increased capacity for energy harvest. *Nature.* (2006) 444:1027–31. 10.1038/nature05414 17183312

[3] Robertson R Manges A Finlay B Prendergast A. The human microbiome and child growth – first 1000 days and beyond. *Trends Microbiol.* (2019) 27:131–47. 10.1016/j.tim.2018.09.008 30529020

[4] Nash M Frank D Friedman J. Early microbes modify immune system development and metabolic homeostasis-the “restaurant” hypothesis revisited. *Front Endocrinol.* (2017) 8:349. 10.3389/fendo.2017.00349 29326657 PMC5733336

[5] Blanton L Charbonneau M Salih T Barratt M Venkatesh S Ilkaveya O Gut bacteria that prevent growth impairments transmitted by microbiota from malnourished children. *Science.* (2016) 351:aad3311-1–3311-7. 10.1126/science.aad3311 26912898 PMC4787260

[6] Younge N Newgard C Cotten C Goldberg R Muehlbauer M Bain J Disrupted maturation of the microbiota and metabolome among extremely preterm infants with postnatal growth failure. *Sci Rep.* (2019) 9:8167. 10.1038/s41598-019-44547-y 31160673 PMC6546715

[7] Kamng’ona A Young R Arnold C Kortekangas E Patson N Jorgensen J The association of gut microbiota characteristics in Malawian infants with growth and inflammation. *Sci Rep.* (2019) 9:12893. 10.1038/s41598-019-49274-y 31501455 PMC6733848

[8] Krebs N Sherlock L Westcott J Culbertson D Hambidge K Feazel L Effects of different complementary feeding regimens on iron status and enteric microbiota in breastfed infants. *J Pediatr.* (2013) 163:416–23. 10.1016/j.jpeds.2013.01.024 23452586 PMC3674183

[9] Tang M Hendricks A Krebs NF. A meat- or dairy-based complementary diet leads to distinct growth patterns in formula-fed infants: a randomized controlled trial. *Am J Clin Nutr.* (2018) 107:734–42. 10.1093/ajcn/nqy038 29722841 PMC6128676

[10] Tang M Andersen V Hendricks A Krebs N. Different growth patterns persist at 24 months of age in formula-fed infants randomized to consume a meat- or dairy-based complementary diet from 5 to 12 months of age. *J Pediatr.* (2019) 206:78–82. 10.1016/j.jpeds.2018.10.020 30413312 PMC6389371

[11] Koletzko B Broekaert I Demmelmair H Franke J Hannibal I Oberle D Protein intake in the first year of life: a risk factor for later obesity? The E.U. childhood obesity project. *Adv Exp Med Biol.* (2005) 569:69–79. 10.1007/1-4020-3535-7_12 16137110

[12] Tang M Weaver N Berman L Brown L Hendricks A Krebs N. Different blood metabolomics profiles in infants consuming a meat- or dairy-based complementary diet. *Nutrients.* (2021) 13:388. 10.3390/nu13020388 33513734 PMC7912106

[13] Hara N Alkanani A Ir D Robertson C Wagner B Frank D Prevention of virus-induced type 1 diabetes with antibiotic therapy. *J Immunol.* (2012) 189:3805–14. 10.4049/jimmunol.1201257 22988033

[14] Markle J Frank D Mortin-Toth S Robertson C Feazel L Rolle-Kampczyk U Sex differences in the gut microbiome drive hormone-dependent regulation of autoimmunity. *Science.* (2013) 339:1084–8. 10.1126/science.1233521 23328391

[15] Frank D. BARCRAWL and BARTAB: software tools for the design and implementation of barcoded primers for highly multiplexed DNA sequencing. *BMC Bioinformatics.* (2009) 10:362. 10.1186/1471-2105-10-362 19874596 PMC2777893

[16] Lane D Pace B Olsen G Stahl D Sogin M Pace N. Rapid determination of 16S ribosomal RNA sequences for phylogenetic analyses. *Proc Natl Acad Sci U.S.A.* (1985) 82:6955–9. 10.1073/pnas.82.20.6955 2413450 PMC391288

[17] Weisburg W Barns S Pelletier D Lane D. 16S ribosomal DNA amplification for phylogenetic study. *J Bacteriol.* (1991) 173:697–703. 10.1128/jb.173.2.697-703.1991 1987160 PMC207061

[18] Illumina. *Homo Sapiens U Hg19 Human Genome Sequence from iGenome.* (2009). Available online at: http://support.illumina.com/sequencing/sequencing_software/igenome.ilmn (accessed August 14, 2014).

[19] Langmead B Salzberg S. Fast gapped-read alignment with Bowtie 2. *Nat Methods.* (2012) 9:357–9. 10.1038/nmeth.1923 22388286 PMC3322381

[20] Frank D Qiu Y Cao Y Zhang S Lu L Kofonow J A dysbiotic microbiome promotes head and neck squamous cell carcinoma. *Oncogene.* (2022) 41:1269–80. 10.1038/s41388-021-02137-1 35087236 PMC8882136

[21] Ewing B Green P. Base-calling of automated sequencer traces using phred. II. Error probabilities. *Genome Res.* (1998) 8:186–94. 9521922

[22] Ewing B Hillier L Wendl M Green P. Base-calling of automated sequencer traces using phred. I. Accuracy assessment. *Genome Res.* (1998) 8:175–85. 10.1101/gr.8.3.175 9521921

[23] Edgar R Haas B Clemente J Quince C Knight R. UCHIME improves sensitivity and speed of chimera detection. *Bioinformatics.* (2011) 27:2194–200. 10.1093/bioinformatics/btr381 21700674 PMC3150044

[24] Schloss P Westcott S. Assessing and improving methods used in operational taxonomic unit-based approaches for 16S rRNA gene sequence analysis. *Appl Environ Microbiol.* (2011) 77:3219–26. 10.1128/AEM.02810-10 21421784 PMC3126452

[25] Pruesse E Peplies J Glockner F. SINA: accurate high throughput multiple sequence alignment of ribosomal RNA genes. *Bioinformatics.* (2012) 28:1823–9. 10.1093/bioinformatics/bts252 22556368 PMC3389763

[26] Quast C Pruesse E Yilmaz P Gerken J Schweer T Yarza P The SILVA ribosomal RNA gene database project: improved data processing and web-based tools. *Nucleic Acids Res.* (2013) 41:D590–6. 10.1093/nar/gks1219 23193283 PMC3531112

[27] Robertson C Harris J Wagner B Granger D Browne K Tatem B Explicet: graphical user interface software for metadata-driven management, analysis and visualization of microbiome data. *Bioinformatics.* (2013) 29:3100–1. 10.1093/bioinformatics/btt526 24021386 PMC3834795

[28] Hale V Jeraldo P Mundy M Yao J Keeney G Scott N Synthesis of multi-omic data and community metabolic models reveals insights into the role of hydrogen sulfide in colon cancer. *Methods.* (2018) 149:59–68. 10.1016/j.ymeth.2018.04.024 29704665 PMC6191348

[29] Moreau N Goupry S Antignac J Monteau F Le Bizec B Champ M Simultaneous measurement of plasma concentrations and 13C-enrichment of short-chain fatty acids, lactic acid and ketone bodies by gas chromatography coupled to mass spectrometry. *J Chromatogr B Analyt Technol Biomed Life Sci.* (2003) 784:395–403. 10.1016/s1570-0232(02)00827-9 12505787

[30] R CoreTeam. *R: A Language and Environment for Statistical Computing.* Vienna: R Foundation for Statistical Computing (2019).

[31] Anderson M Crist T Chase J Vellend M Inouye B Freestone A Navigating the multiple meanings of beta diversity: a roadmap for the practicing ecologist. *Ecol Lett.* (2011) 14:19–28. 10.1111/j.1461-0248.2010.01552.x 21070562

[32] Oksanen J Blanchet G Friendly M Kindt R Legendre P McGlinn D *Vegan: Community Ecology Package. R Package Version 2.5-7.* (2019).

[33] Bates D Maechler M Bolker B Walker S. Fitting linearmmixed-effects models using lme4. *J Stat Softw.* (2015) 67:1–48.

[34] Fernandes A Macklaim J Linn T Reid G Gloor G. ANOVA-like differential expression (ALDEx) analysis for mixed population RNA-Seq. *PLoS One.* (2013) 8:e67019. 10.1371/journal.pone.0067019 23843979 PMC3699591

[35] Fernandes A Reid J Macklaim J McMurrough T Edgell D Gloor G. Unifying the analysis of high-throughput sequencing datasets: characterizing RNA-seq, 16S rRNA gene sequencing and selective growth experiments by compositional data analysis. *Microbiome.* (2014) 2:15. 10.1186/2049-2618-2-15 24910773 PMC4030730

[36] Laursen M Andersen L Michaelsen K Molgaard C Trolle E Bahl M Infant gut microbiota development is driven by transition to family foods independent of maternal obesity. *mSphere.* (2016) 1:e00069-15. 10.1128/mSphere.00069-15 27303699 PMC4863607

[37] Qasem W Azad M Hossain Z Azad E Jorgensen S Castillo San Juan S Assessment of complementary feeding of Canadian infants: effects on microbiome & oxidative stress, a randomized controlled trial. *BMC Pediatr.* (2017) 17:54. 10.1186/s12887-017-0805-0 28196533 PMC5310014

[38] Forbes J Azad M Vehling L Tun H Konya T Guttman D Canadian healthy infant longitudinal development study i. association of exposure to formula in the hospital and subsequent infant feeding practices with gut microbiota and risk of overweight in the first year of life. *JAMA Pediatr.* (2018) 172:e181161. 10.1001/jamapediatrics.2018.1161 29868719 PMC6137517

[39] Ma J Li Z Zhang W Zhang C Zhang Y Mei H Comparison of gut microbiota in exclusively breast-fed and formula-fed babies: a study of 91 term infants. *Sci Rep.* (2020) 10:15792. 10.1038/s41598-020-72635-x 32978424 PMC7519658

[40] Azad M Konya T Maughan H Guttman D Field C Chari R Gut microbiota of healthy Canadian infants: profiles by mode of delivery and infant diet at 4 months. *CMAJ.* (2013) 185:385–94. 10.1503/cmaj.121189 23401405 PMC3602254

[41] Hill C Lynch D Murphy K Ulaszewska M Jeffery I O’Shea C Evolution of gut microbiota composition from birth to 24 weeks in the INFANTMET Cohort. *Microbiome.* (2017) 5:4. 10.1186/s40168-016-0213-y 28095889 PMC5240274

[42] Karcher N Nigro E Puncochar M Blanco-Miguez A Ciciani M Manghi P Genomic diversity and ecology of human-associated *Akkermansia* species in the gut microbiome revealed by extensive metagenomic assembly. *Genome Biol.* (2021) 22:209. 10.1186/s13059-021-02427-7 34261503 PMC8278651

[43] Cani P Depommier C Derrien M Everard A de Vos W. *Akkermansia muciniphila*: paradigm for next-generation beneficial microorganisms. *Nat Rev Gastroenterol Hepatol.* (2022) 19:625–37. 10.1038/s41575-022-00631-9 35641786

[44] Alcazar M Escribano J Ferré N Closa-Monasterolo R Selma-Royo M Feliu A Gut microbiota is associated with metabolic health in children with obesity. *Clin Nutr.* (2022) 41:1680–8. 35777107 10.1016/j.clnu.2022.06.007

[45] Reyna M Petersen C Dai D Dai R Becker A Azad M Longitudinal body mass index trajectories at preschool age: children with rapid growth have differential composition of the gut microbiota in the first year of life. *Int J Obes.* (2022) 46:1351–8. 10.1038/s41366-022-01117-z 35428865 PMC9239911

[46] Zhu Y Lin X Zhao F Shi X Li H Li Y Meat, dairy and plant proteins alter bacterial composition of rat gut bacteria. *Sci Rep.* (2015) 5:15220. 10.1038/srep15220 26463271 PMC4604471

[47] Nilsen M Madelen Saunders C Leena Angell I Arntzen M Lodrup Carlsen K Carlsen K Butyrate levels in the transition from an infant- to an adult-like gut microbiota correlate with bacterial networks associated with *Eubacterium rectale* and *Ruminococcus gnavus*. *Genes.* (2020) 11:1245. 10.3390/genes11111245 33105702 PMC7690385

[48] Cummings J Macfarlane G. The control and consequences of bacterial fermentation in the human colon. *J Appl Bacteriol.* (1991) 70:443–59. 1938669 10.1111/j.1365-2672.1991.tb02739.x

[49] Millward D Layman D Tome D Schaafsma G. Protein quality assessment: impact of expanding understanding of protein and amino acid needs for optimal health. *Am J Clin Nutr.* (2008) 87:1576S–81S. 10.1093/ajcn/87.5.1576S 18469291

[50] Rasmussen H Holtug K Mortensen P. Degradation of amino acids to short-chain fatty acids in humans. An in vitro study. *Scand J Gastroenterol.* (1988) 23:178–82. 10.3109/00365528809103964 3363290

