# Supplementary material for: Different gut microbiota in U.S. formula-fed infants consuming a meat vs. dairy-based complementary foods: A randomized controlled trial
Source: Front Nutr. 2023 Jan 26;9:1063518. doi: 10.3389/fnut.2022.1063518 (PMC9909089; doi:10.3389/fnut.2022.1063518)
Supplement: Supplementary file 1 [file Data_Sheet_1.docx]

**Supplementary Figure 1**


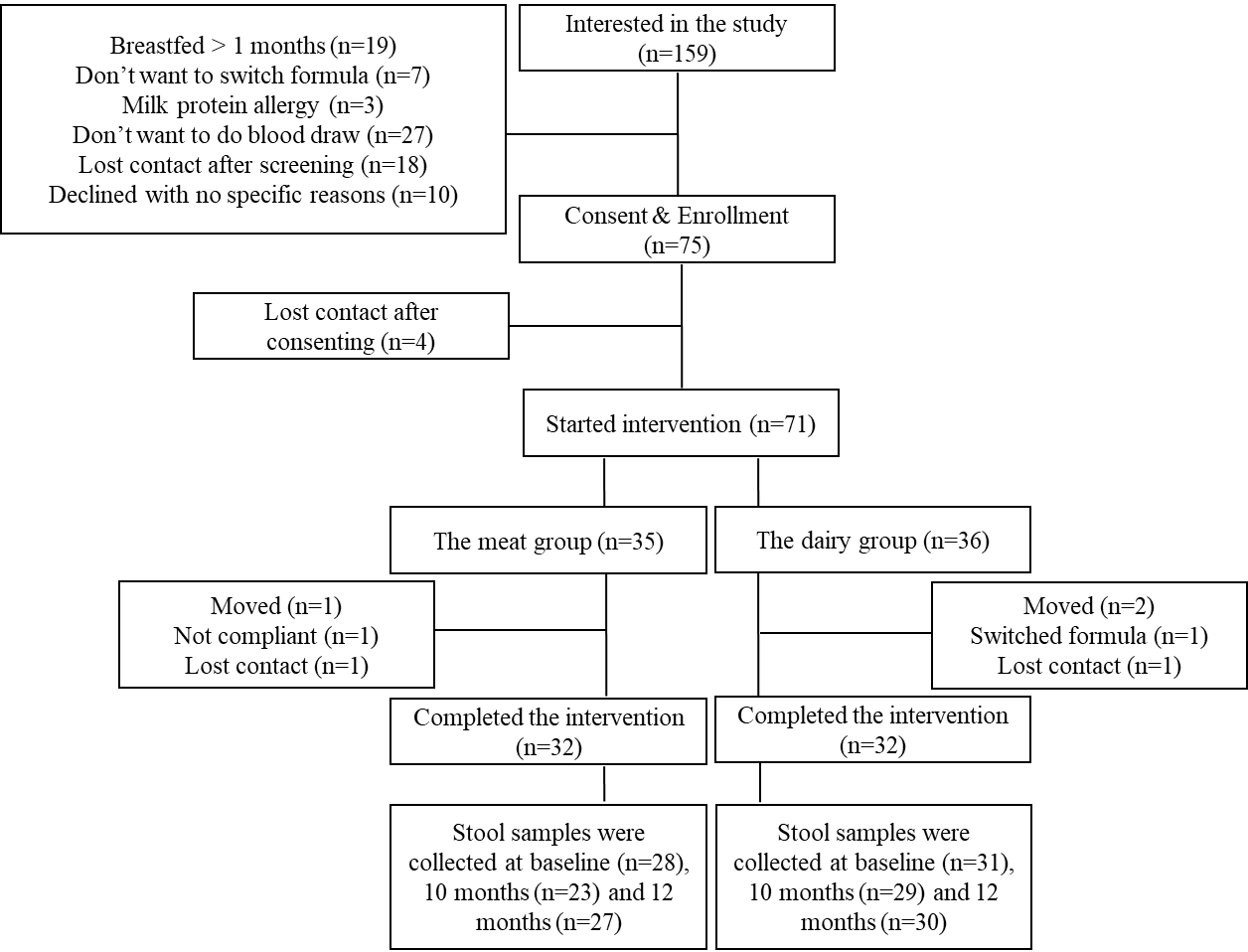


**Supplemental Figure 2. Individual taxa with significant changes in abundance with age.** The development of individual taxa with age was evaluated through linear mixed effects modeling of centered log-ratio (CLR) transformed sequence count data, as described in the text. Plots show the development of taxa that had significant associations with age (FDR < 0.1; see **Figure 4A**) after adjusting for Diet group. The x-axes and y-axes specify infant age in months and predicted CLR values following linear mixed effects modeling. Lines are fitted values from linear models, and shaded ribbons indicate 95% confidence intervals. Circles and triangles represent mean values for each Diet group and age. Plots are listed (left to right, top to bottom) in descending order of mean relative abundance across all infants. Taxa names are preceded by abbreviated phylum names: Bact = Bacteroidetes. Firm = Firmicutes. Prot = Proteobacteria.

**
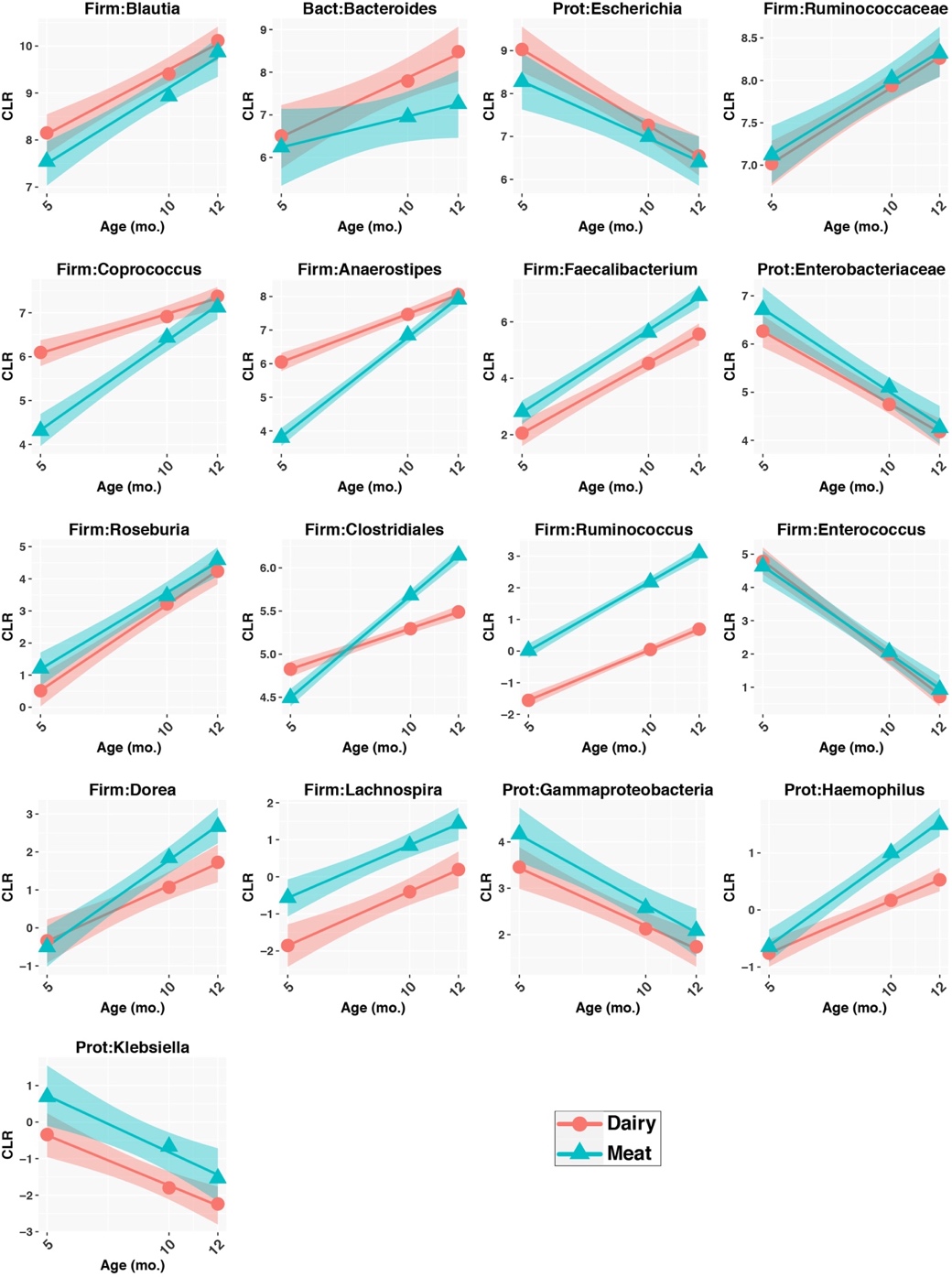
**
